# Attenuation of neuronal ferroptosis in intracerebral hemorrhage by inhibiting HDAC1/2: Microglial heterogenization via the Nrf2/HO1 pathway

**DOI:** 10.1111/cns.14646

**Published:** 2024-03-24

**Authors:** Zhiwen Jiang, Heng Yang, Wei Ni, Xinjie Gao, Xu Pei, Hanqiang Jiang, Jiabin Su, Ruiyuan Weng, Yuchao Fei, Yanqin Gao, Yuxiang Gu

**Affiliations:** ^1^ Department of Neurosurgery of Huashan Hospital, State Key Laboratory of Medical Neurobiology and MOE Frontiers Center for Brain Science, Institutes of Brain Science Fudan University Shanghai China

**Keywords:** ferroptosis, HDACs, intracerebral hemorrhage, Nrf2, Romidepsin

## Abstract

**Aim:**

The class I histone deacetylases (HDACs) implicate in microglial heterogenization and neuroinflammation following Intracerebral hemorrhage (ICH). Ferroptosis has also been reported in the ICH model. However, the relationship between HDAC1/2's role in microglial heterogenization and neuronal ferroptosis remains unclear.

**Methods:**

In both in vivo and in vitro models of ICH, we used Romidepsin (FK228), a selective HDAC1/2 inhibitor, to investigate its effects on microglial heterogenization and neuronal ferroptosis. In the in vitro ICH model using Hemin, a transwell system was utilized to examine how microglia‐driven inflammation and ICH‐triggered neuronal ferroptosis interact. Immunostaining, Western blotting and RT‐qPCR were used to evaluate the microglial heterogenization and neuronal ferroptosis. Microglial heterogenization, neuronal ferroptosis, and neurological dysfunctions were assessed in vivo ICH mice model performed by autologous blood injection.

**Results:**

HDAC1/2 inhibition altered microglial heterogenization after ICH, as showing the reducing neuroinflammation and shifting microglia towards an anti‐inflammatory phenotype by immunostaining and qPCR results. HDAC1/2 inhibition reduced ferroptosis, characterized by high ROS and low GPx4 expression in HT22 cells, and reduced iron and lipid deposition post‐ICH in vivo. Additionally, the Nrf2/HO1 signaling pathway, especially acetyl‐Nrf2, activated in the in vivo ICH model due to HDAC1/2 inhibition, plays a role in regulating microglial heterogenization. Furthermore, HDAC1/2 inhibition improved sensorimotor and histological outcomes post‐ICH, offering a potential mechanism against ICH.

**Conclusion:**

Inhibition of HDAC1/2 reduces neuro‐ferroptosis by modifying the heterogeneity of microglia via the Nrf2/HO1 pathway, with a particular focus on acetyl‐Nrf2. Additionally, this inhibition aids in the faster removal of hematomas and lessens prolonged neurological impairments, indicating novel approach for treating ICH.

## Graphical abstract

Administration of HDAC1/2 inhibitor modulates microglial heterogenization towards an anti‐inflammatory phenotype, facilitated by the activation of the Nrf2/HO1 signaling pathway, therefore decreases the ferroptosis in neurons, characterized by reduced ROS and preserved GPX4 activity. Consequently, HDAC1/2 inhibition fosters the clearance of hematoma, mitigates white matter injury, and preserves neurological function.

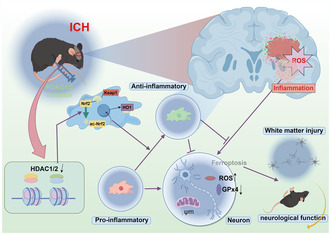

## 1 INTRODUCTION

Intracerebral hemorrhage (ICH), representing 27.9% of all stroke cases, has experienced an increasing trend since 1990.^1^ Research points to substances such as heme, a by‐product of hemoglobin breakdown, as significant factors in brain damage following ICH.^2^, ^3^, ^4^ Heme oxygenase (HO) processes heme into iron, carbon monoxide, and biliverdin. Iron, particularly when labile, is instrumental in generating lipid reactive oxygen species (ROS), leading to tissue damage.^2^, ^5^, ^6^

Iron is key in cell life and death, notably affecting ferroptosis.^7^ Ferroptosis, an iron‐dependent non‐apoptotic cell death form, has been observed in cerebrovascular disease models, including ICH, traumatic brain injury (TBI), and middle cerebral artery occlusion (MCAO).^8^, ^9^, ^10^, ^11^ Characterized by lethal lipid ROS, mitochondrial morphology changes, and uncontrolled lipid peroxidation, ferroptosis depends on iron‐driven processes.^7^, ^12^ Mitochondria, central in oxidative metabolism, play a significant role in ferroptosis, evident from specific mitochondrial changes during ferroptosis.^13^

Microglia, the brain's primary immune responders, exhibit various phenotypes, notably the pro‐inflammatory and the anti‐inflammatory.^14^, ^15^, ^16^ The pro‐inflammatory phenotype exacerbates neurological deficits post‐ICH through harmful cytokine and ROS production.^17^, ^18^ Conversely, The anti‐inflammatory phenotype helps in post‐ICH recovery by producing anti‐inflammatory factors and accelerating the removal of tissue debris and cell regeneration.^19^ Hence, therapies focusing on reducing inflammation and clearing debris like hemoglobin and iron show potential for ICH.

Histone deacetylases (HDACs), key in chromatin remodeling, are categorized into four classes, with HDAC1, HDAC2, HDAC3, and HDAC8 in Class I.^20^ HDACs and their inhibitors (HDACi) have exhibited neuroprotective properties and the ability to modulate microglia/macrophage heterogenization in cerebrovascular disease models, including ICH, TBI, and ischemic stroke.^21^, ^22^, ^23^, ^24^, ^25^ HDAC1 and HDAC2, particularly abundant in microglia/macrophages, respond significantly to LPS stimulation.^26^ Previous study confirmed that HDAC2 knockout can modulate microglial heterogenization and alleviate neurological deficits post‐ICH.^21^ HDAC1 and HDAC2, being highly similar, can substitute for each other's roles. Blocking just one of HDAC1/2 in macrophages boosts the other's activity, thus simultaneously inhibiting the activity of both can reduce LPS‐caused inflammation.^27^ Research shows that HDAC1/2 jointly regulate microglial function, impacting disease progression. Eliminating HDAC1/2 in microglia reduces inflammation, enhances amyloid plaque clearance, and boosts cognition in Alzheimer's mice.^28^ Additionally, HDAC2 participated in the ferroptosis and neuroinflammation in cauda equina injury and which were inhibited by (Valproic Acid) VPA.^29^

The transcription factor, Neuclear factor erythroid 2‐related factor 2 (Nrf2), is crucial in protecting cells from ferroptosis, mainly through triggering antioxidant responses and regulating iron metabolism.^30^, ^31^, ^32^, ^33^, ^34^, ^35^ Nrf2 is crucial in protecting cells from ferroptosis, mainly through triggering antioxidant responses and regulating iron metabolism.^36^ Furthermore, the stability of Nrf2 is bolstered through acetylation, which enhances its translocation to the nucleus and amplifies its anti‐inflammatory efficacy.^37^, ^38^

Romidepsin (FK228), an FDA‐approved selective inhibitor of HDAC1/2,^39^ shifts microglial heterogeneity towards an anti‐inflammatory phenotype and exhibits neuroprotective properties.^40^ The link between HDACs and ferroptosis is established, with studies indicating that HDAC inhibitors block ferroptosis and offer neuroprotection.^11^, ^41^ Therefore, we hypothesize that HDAC1/2 inhibition lies in its ability to promote microglial heterogenization, thereby reducing neuroinflammation, inhibiting neuronal ferroptosis, and enhancing neuronal survival, potentially leading to novel ICH treatment strategies.

## 2 METHOD

### 2.1 Experimental animals

C57BL/6 mice were obtained from the Slac Laboratory Animals Center (Shanghai, China). All experimental protocols were approved by the Department of Laboratory Animal Science, Fudan University (approve number: 2921JS Huashan hospital‐325). The mice were provided with free access to food and water.

### 2.2 The in vivo ICH model and administration of Romidepsin

Intracerebral injections were administered according to previously established protocols.^42^ Mice were anesthetized with isoflurane and positioned in a stereotaxic frame. Following the creation of a burr hole in the skull, 30 μL of the subject's own blood was infused into the right basal ganglia at a flow rate of 2 μL/min. The needle was kept in position for 10 min post‐injection to prevent backflow. Post‐procedure, the hole was sealed and the incision sutured. Sham‐operated mice received the same procedure minus the injection. The HDAC1/2 inhibitor Romidepsin (FK228) was administered intraperitoneally at 0.5 mg/kg immediately after ICH and biweekly for 2 weeks, while controls received a 10% DMSO injection.^40^

### 2.3 The in vitro ICH model

For in vitro model, BV2 and HT22 was cultured in a transwell system, Hemin (20 uM)^43^ and FK228 (10 ng/mL)^44^ were added into the system for 24 h. The following assays were performed 24 h later: LDH activity assay for loss of membrane integrity, RT‐qPCR for FTH1, ACSL4, GPx4 in HT22 and for CD16, TNFα, IL6, CD206, TGF‐β, IL10 in BV2, and immunocytochemical staining for ROS, Lipid drop, GPx4 in HT22 and for CD16 and CD206 in BV2, and western blot for GPx4 in HT22 were performed 24 h later.

### 2.4 Magnetic resonance imaging (MRI)

11.7T MRI system (Bruker BioSpec, Karlsruhe, Germany), at the Institute of Science and Technology and Brain‐inspired Intelligence in Shanghai of China, was employed to scan the mice brain on days 1, 3, and 7 following ICH. During the 11.7T MRI scans, mice were kept anesthetized with a 3% isoflurane/air mixture. The imaging protocol for all the mice included T2*‐weighted (repetition time/echo time = 4000/60 ms; slice thickness = 0.8 mm) and SWI sequences (repetition time/echo time = 250/5 ms; slice thickness = 0.5 mm). The field of view was 20 × 20 mm, and the matrix was 256 × 256 mm. A total of 25 coronal slices with a thickness of 0.5 mm were obtained, covering the area from the frontal pole to the brain stem. Afterwards, the SWI lesion volume was calculated by ImageJ software (National Institutes of Health, Bethesda, MD, USA). The SWI lesion, indicative of hematoma size on days 1, 3, and 7, was demarcated along the edge of the low‐intensity region. The total lesion volume was calculated by aggregating the low‐intensity areas across all slices. The T2* mapping values were assessed for three distinct zones surrounding the hematoma and their respective mirror regions in the opposite hemisphere, showing as a percentage of ipsilateral / contralateral×100%.

### 2.5 Behavioral tests

Behavioral tests were conducted by a researcher blinded to the experimental conditions. Sensorimotor function assessments, using the rotarod test, foot fault test, and wire hanging test, were performed from 1 to 28 days after ICH. Before performing the behavioral tests, all animals underwent behavior training for 3 days, and those displaying abnormal behavior were excluded.

### 2.6 Immunofluorescence

Brain sections were immunohistochemically stained for, and densitometric analysis was performed as previously described (Supplementary Methods).^45^

### 2.7 Perl's blue stain and Oil red O assay

Perls' staining was used to detect iron accumulation as previously described.^46^ Oil Red O staining was performed to assess lipid deposition in the peri‐hematoma area.^8^, ^47^ (Supplementary Methods).

### 2.8 FJC staining

FJC staining was performed using the FJC Ready‐to‐Dilute Staining Kit (Biosensis, USA) according to manufacturer's protocol. The brain sections were observed and photographed under a fluorescence microscope at an excitation wavelength of 450–490 nm.

### 2.9 TUNEL assay

Brain sections, fixed in 4% paraformaldehyde and dewaxed, underwent TUNEL assay (Beyotime Biotechnology, China) and were examined with fluorescence microscopy.

### 2.10 Measurement of mitochondrial membrane potential (Δψm)

This study used the Tissue Mitochondria Isolation Kit (C3606, Beyotime) and Enhanced Mitochondrial Membrane Potential Assay Kit with JC‐1 (C2003S, Beyotime) for mitochondrial isolation and fluorescence analysis. Fluorescence microplate reader settings were 490 nm excitation/530 nm emission for JC‐1 monomers, and 525 nm excitation/590 nm emission for JC‐1 aggregates.

### 2.11 Transmission electron microscopy (TEM)

TEM was performed as described previously.^21^ Mice were perfused with saline and ice‐cold 4% paraformaldehyde with 0.1% glutaraldehyde. Tissue around the hematoma was excised, fixed in 2% glutaraldehyde, postfixed with osmium tetroxide, dehydrated in acetone, embedded in resin, and sectioned into 60–90 nm slices. These were stained with uranyl acetate and lead citrate and examined using a JEOL JEM‐1230 transmission electron microscope (JEOL Ltd, Tokyo, Japan).

### 2.12 RNA isolation and quantitation

RNA was extracted from tissues and cells around the hematoma using TRIzol reagents (T2210; Solarbio), following the manufacturer's guidelines. The RNA concentration was measured using a spectrophotometer. Reverse transcription was then conducted using Takara's Reverse Transcription Kit (Japan) in a PCR machine, also as per manufacturer's instructions. Subsequently, RT‐qPCR was performed using the SYBR‐Green method and a fluorescence qPCR detection system. Glyceraldehyde‐3‐phosphate dehydrogenase was used as the internal control for these experiments. Primer sequences can be found in Table S1.

### 2.13 Western blotting

Cellular protein was extracted and quantified using the bicinchoninic acid method. Afterward, 40 μg of protein was subjected to electrophoresis and transferred onto membranes. The membranes were then blocked with 5% bovine serum albumin for 1 h and underwent overnight incubation at 4°C with rabbit primary antibodies against Acetyl‐Histone H4 (1:1000, 06‐866; MilliporeSigma), GPx4 (1:400, ab125066; Abcam), Keap‐1 (1:1000, 10503‐2‐AP; Protein‐tech), Nrf2 (1:1000 16369‐1‐AP, Protein‐tech), HO‐1 (1:1000 16369‐1‐AP, Protein‐tech), and β‐actin (1:1000, ab179467; Abcam). The next day, the membranes were inducted again with rabbit secondary antibodies (1:1000, SE238; Solarbio) at 37°C for 1 h, followed by development using enhanced chemiluminescence reagent.

### 2.14 Statistical analysis

Statistical analysis was conducted using GraphPad Prism 9.0 (GraphPad Software Inc., La Jolla, CA, USA). For multiple Gaussian‐distributed groups, one‐way ANOVA followed by Bonferroni's test was applied. Non‐normal multiple groups were analyzed using the Kruskal–Wallis test. Repeated measurements in groups were evaluated using two‐way ANOVA with Bonferroni's test. Results are shown as mean ± SD. *p* < 0.05 was considered statistically significant.

## 3 RESULTS

### 3.1 Inhibition of HDAC1/2 promotes histone acetylation and modulates the microglial heterogenization in the in vitro ICH model

Given that deletion of HDAC1/2 has been shown to reduce neuroinflammation mediated by microglia and improve neurological conditions in Alzheimer's disease mouse models,^28^ our initial step was to determine if inhibiting HDAC1/2 could similarly influence microglial heterogeneity and neuroinflammation in an in vitro model of ICH.

**FIGURE 1:**
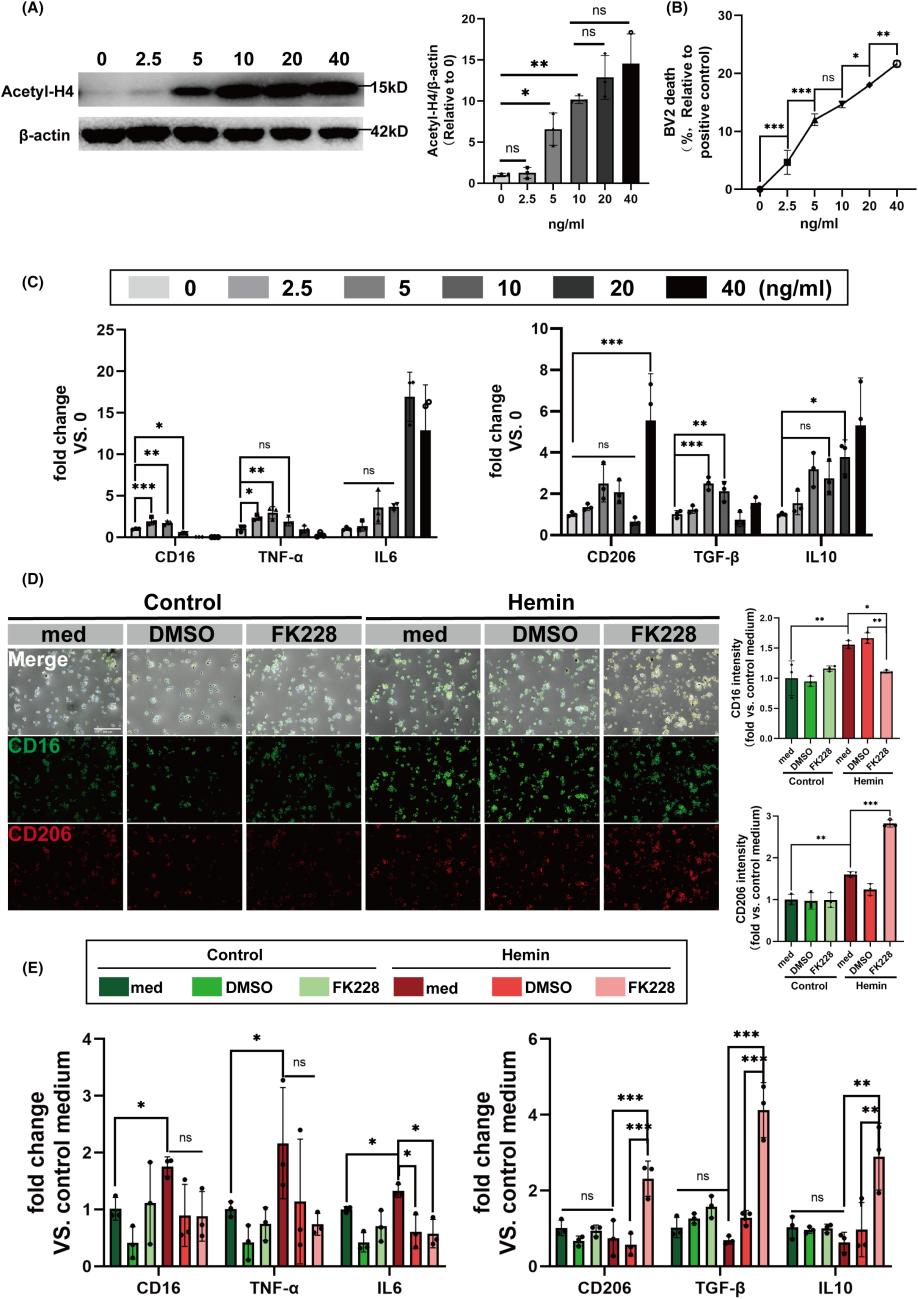
HDAC1/2 inhibition modulates the heterogenization of microglia in vitro. (A) The acetyl‐H4 level of BV2 was quantified by western blotting after 24 h of exposure to varying concentrations of FK228 (0, 2.5, 5, 10, 20, 40 ng/mL). (B) LDH release in BV2 cells was quantified after 24 h of exposure to varying concentrations of FK228 (0, 2.5, 5, 10, 20, 40 ng/mL). (C) The mRNA expression levels of CD16, IL‐6, TNF‐α, CD206, TGF‐β and IL‐10 as quantified by RT‐qPCR after 24 h of exposure to varying concentrations of FK228 (0, 2.5, 5, 10, 20, 40 ng/mL). (D) Representative immunofluorescence images of CD16 (green)/CD206 (red)‐positive BV2 (scale bar = 300 μm). (E) Quantitative analysis of the mRNA expression levels of CD16, IL‐6, TNF‐α, CD206, TGF‐β and IL‐10 in BV2 treated with vehicle or FK228. All the data are presented as the mean ± SD. One‐way ANOVA test and Bonferroni post hoc (A–E). *n* = 3 per group, *p < 0.05, **p < 0.01, ***p < 0.001, ns: no significance.

Firstly, we monitored histone H4 acetylation levels as a biomarker for HDAC1/2 inhibition efficacy (Figure 1A).^48^, ^49^, ^50^ Concomitantly, BV2 cells subjected to varying concentrations of FK228 (0, 2.5, 5, 10, 20, 40 ng/mL), with cellular viability quantified through LDH release assays. Our data revealed a dose‐dependent increase in acetyl‐histone H4, with a concentration of 10 ng/mL FK228 notably enhancing acetylation, while concurrently maintaining low cytotoxicity (Figure 1B). Intriguingly, at this concentration, FK228 appeared to induce an anti‐inflammatory phenotype in resting microglia (Figure 1C). Subsequently, we treated HT22 cells with varying concentrations of Hemin to establish in vitro ICH model. Following Hemin simulation, the level of ROS was upregulated (Figure S1D). Then, we evaluated LDH release, ROS levels, and ferroptosis‐specific phenotypic changes. Our findings identified 20 μM as the optimal Hemin concentration for the in vitro ICH model (Figure S1A–C).

We further investigated the effects of FK228 on heterogenization of microglia in this in vitro ICH model. Employing a combination of immunostaining and RT‐qPCR techniques, we assessed the expression of microglial heterogenization markers post‐treatment. Notably, FK228‐treated BV2 exhibited a marked decrease in CD16 protein levels, concomitant with an upregulation of CD206 expression (Figure 1D). The alterations were corroborated by RT‐qPCR analysis, which revealed transcriptional changes consistent with the protein expression patterns we noted (Figure 1E).

### 3.2 Inhibition of HDAC1/2 mitigates the neuronal ferroptosis by modulating the microglial heterogenization in the in vitro ICH model

Previous research has shown that targeting the reduction of neuroinflammation can inhibit neuronal ferroptosis.^51^ Therefore, to elucidate the potential of FK228 in modulating microglial influences on Hemin‐induced neuronal ferroptosis, we incorporated FK228 or a corresponding vehicle into a transwell system.

Given the critical role of lipid peroxidation in ferroptosis, we meticulously examined the normal lipid profile, lipid peroxidation‐specific ROS, and the involvement of glutathione peroxidase 4 (GPx4) in the lipid peroxidation process within HT22 cells.

**FIGURE 2:**
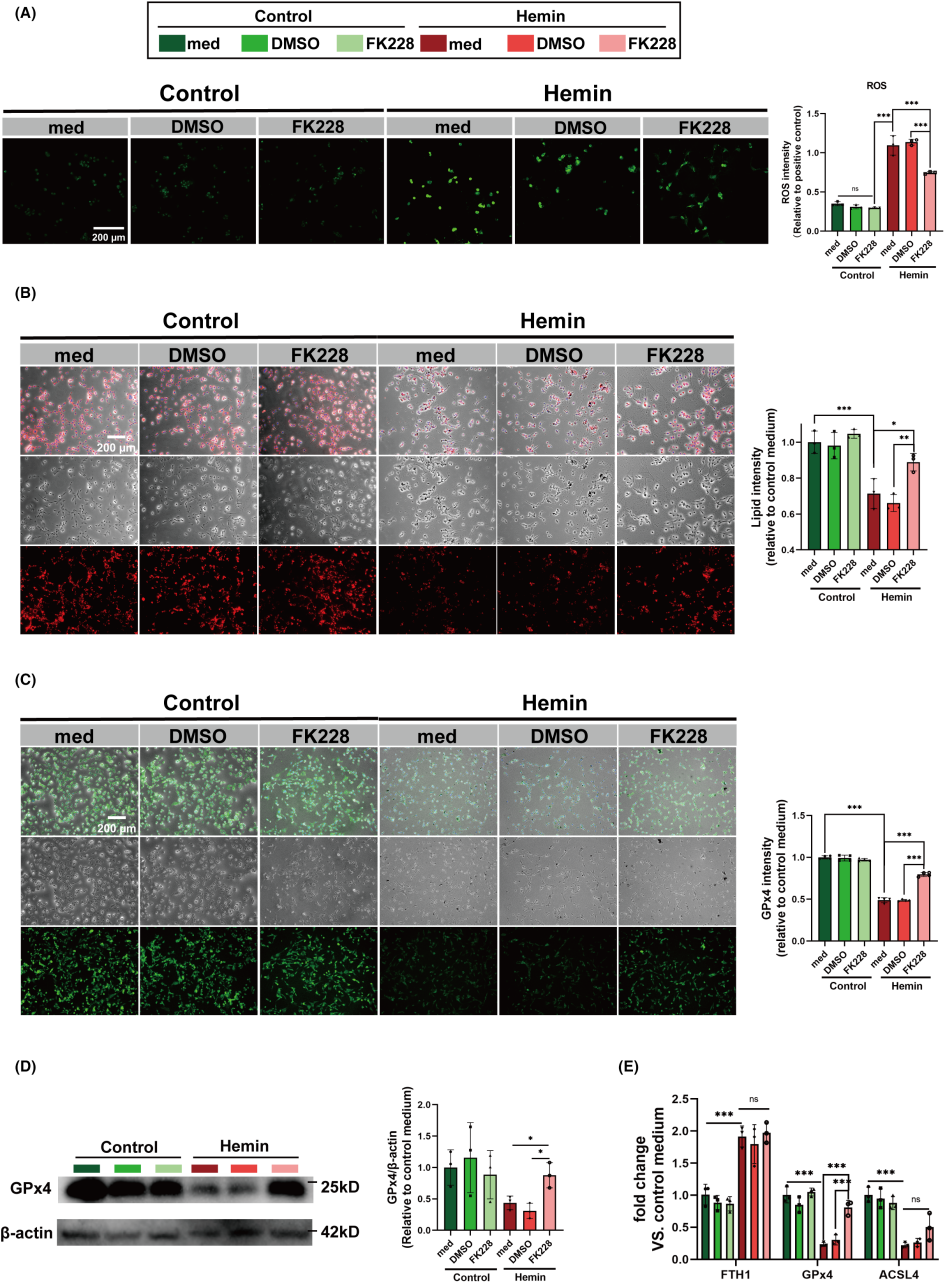
HDAC1/2 inhibition mitigates the neuronal ferroptosis by modulating microglial heterogenization in vitro ICH model. (A) ROS levels in HT22 cells treated with FK228 or vehicle (DMSO) after Hemin‐induced model (scale bar = 200 μm). (B) Representative images of lipid droplet staining in HT22 cells with varying treatments, providing insight into lipid metabolism changes post‐Hemin exposure (scale bar = 200 μm). (C) Representative images of immunofluorescence staining for GPx4 in HT22 cells (scale bar = 200 μm). (D) Western blotting analysis of GPx4 protein levels in HT22 cells. (E) The mRNA expression levels of FTH1, GPx4, ACSL4 as quantified by RT‐qPCR after treatment with Hemin and FK228. All the data are presented as the mean ± SD. One‐way ANOVA test and Bonferroni post hoc (A–E). *n* = 3 per group (A, D, E), *n* = 4 per group (B, C), *p < 0.05, **p < 0.01, ***p < 0.001, ns: no significance.

FK228 effectively suppressed the elevated ROS levels induced by Hemin (Figure 2A). Additionally, the lipid immunostaining data revealed a notable reduction in lipid content in HT22 cells subjected to Hemin induction, a phenomenon that was ameliorated upon FK228 treatment (Figure 2B). The expression level of GPx4 in HT22 cells also was elevated by the FK228 treatment after the Hemin induction (Figure 2C). Complementing these findings, western blotting analyses of HT22 cells demonstrated that FK228 treatment significantly upregulated GPx4 expression (Figure 2D). This upregulation was further validated by RT‐qPCR, aligning with the protein results (Figure 2E). In conclusion, our study showed that mitigating the neuroinflammation triggered by microglia can effectively decrease ferroptosis in HT22 cells.

### 3.3 Inhibition of HDAC1/2 reduces neuronal ferroptosis and degeneration in the in vivo ICH model

Then we detected the influence of HDAC1/2 inhibition in neuronal vitality and ferroptosis characteristics following ICH. Ferroptosis, characterized by lipid peroxidation induced by iron overload, prompted us to explore FK228's impact on iron and lipid deposition. Following ICH, the obvious iron and lipid droplets deposition were observed in ICH mice (Figure S2A).

**FIGURE 3:**
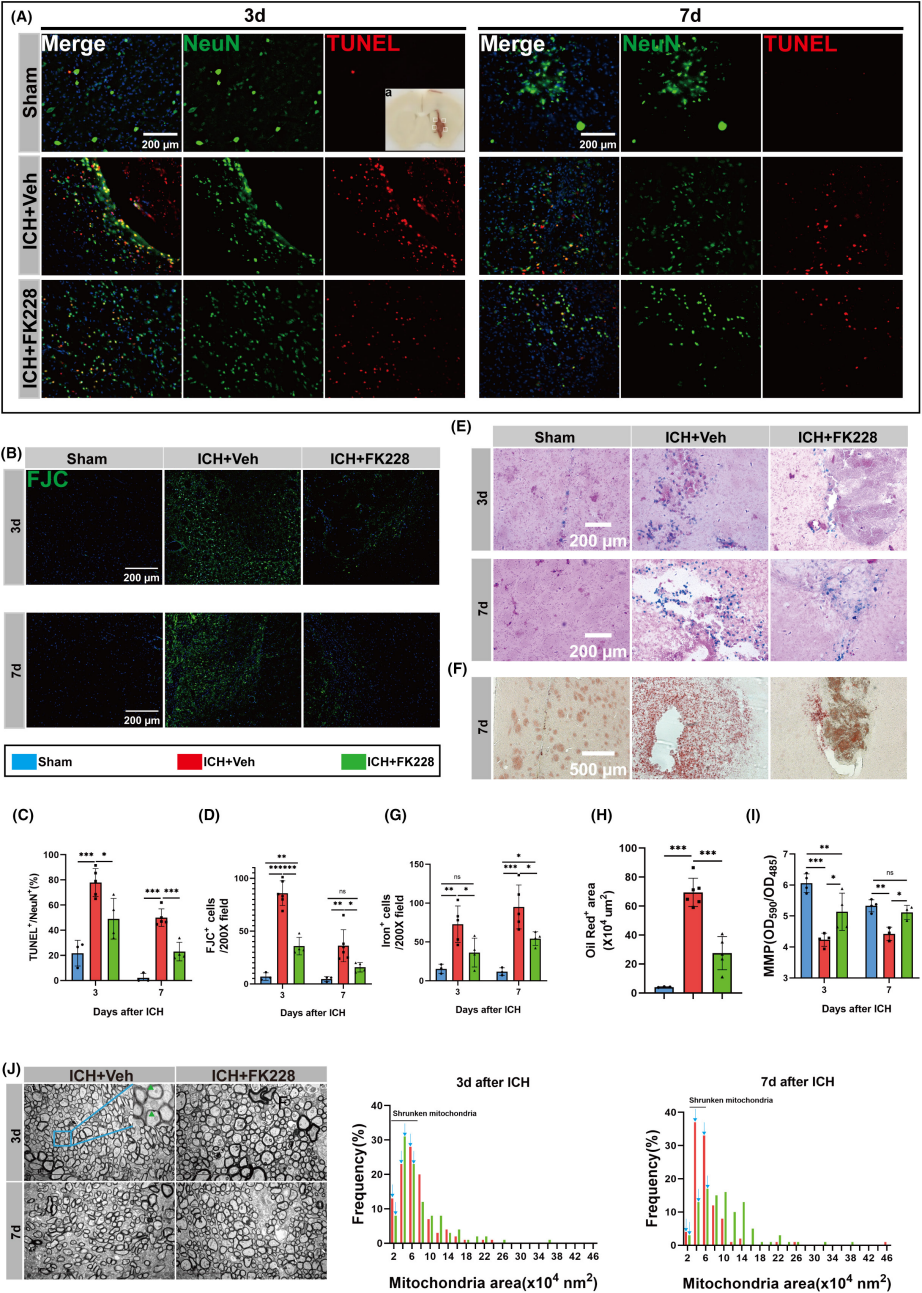
HDAC1/2 inhibition reduces neuronal ferroptosis and degeneration in vivo ICH model. (A) Sampling location diagram. (A, C) TUNEL staining was used to mark apoptosis neurons at day 3 and 7 after ICH. Representative images and quantification are shown. *n* = 3 for Sham; *n* = 4 for ICH and Vehicle, *n* = 4 for ICH and FK228. (scale bar = 200 μm). (B, D) FJC staining at day 3 and 7 after ICH. Representative images and quantification are shown. *n* = 3 for Sham; *n* = 4 for ICH and Vehicle, *n* = 4 for ICH and FK228. (scale bar = 200 μm). (E, G) Representative image of Perls' staining and quantification of iron‐positive cells. *n* = 3 for Sham; *n* = 5 for ICH and Vehicle, *n* = 4 for ICH and FK228. (scale bar = 200 μm). (F, H) Representative image of Oil red O staining and quantification of Oil red O‐positive area. *n* = 3 for Sham; *n* = 5 for ICH and Vehicle, *n* = 4 for ICH and FK228. (scale bar = 500 μm). (I) Mitochondria membrane potential (MMP) of tissue around the hematoma at 3 and 7 days for mice after ICH. 3d, *n* = 4 for Sham; *n* = 4 for ICH and Vehicle, *n* = 4 for ICH and FK228; 7d, *n* = 4 for Sham; *n* = 3 for ICH and Vehicle, *n* = 3 for ICH and FK228. (J) Ultrastructure of neuron axons and quantification analysis of the mitochondrial area frequency in axons on the 3 and 7 days post‐ICH. *n* = 3 for ICH and Vehicle, *n* = 3 for ICH and FK228. Number of axon mitochondria, 3d, *n* = 194 for ICH and Vehicle, *n* = 181 for ICH and FK228 ICH and Vehicle; 7d, *n* = 134 for ICH and vehicle, *n* = 165 for ICH and FK228. Shrunken mitochondrial can be seen (green borrow). All the data are presented as the mean ± SD. One‐way ANOVA test and Bonferroni post hoc (C, D, G–I). *p < 0.05, **p < 0.01, ***p < 0.001, ns: no significance.

Brain sections from mice at 3, 7 days post‐ICH showed reduced neuronal death and increased surviving neurons in the FK228‐treated group, confirmed by TUNEL (Figure 3A,C) and FJC staining (Figure 3B,D). Additionally, our research indicated that administering FK228 notably reduced the quantity of iron‐rich hematoxylin in cells compared to the group that did not receive treatment (Figure 3E,G). Similarly, the feature of lipid metabolism was detected. Oil Red O staining was performed on the 1st, 3rd, and 7th days after ICH. The obvious lipid droplets were only detected until the 7th day post‐ICH (Figure S2A). Notably, FK228 treatment led to a reduction in the deposition area of lipid droplets (Figure 3F,H). Then, we thoroughly examined the structure and function of mitochondria, given their well‐established role in the regulation of ferroptosis.^52^ To further probe mitochondrial integrity, we evaluated mitochondrial membrane potential (MMP). Post‐ICH, a marked decrease in this potential was evident, signifying mitochondrial impairment. FK228 administration attenuated this decline (Figure 3I), hinting at its protective influence on mitochondrial function. Further, TEM was employed to scrutinize cellular ultrastructure alterations following ICH. We prepared TEM sections from mice at 3 and 7 days post‐ICH. Notably, at both time points, we observed a significant prevalence of shrunken mitochondria in axons at the hematoma margin (Figure 3J). Overall, FK228 mitigated ICH‐related iron and lipid metabolic disturbances, reducing neuronal death.

### 3.4 Inhibition of HDAC1/2 modulates microglial heterogenization and restrains inflammation via Nrf2 acetylation in the in vivo ICH model

**FIGURE 4:**
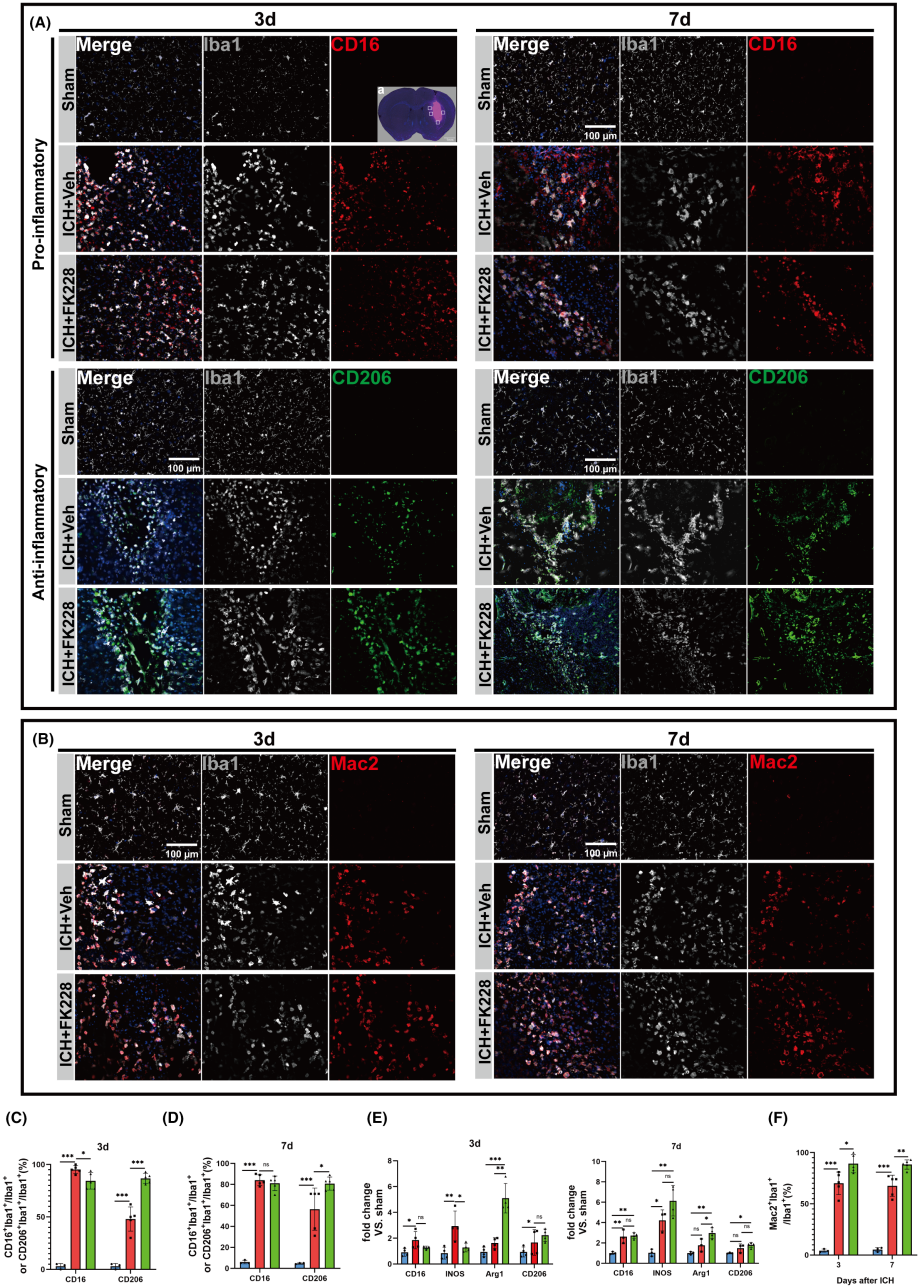
HDAC1/2 inhibition modulates the heterogenization of microglia in vivo ICH model. (A) Sampling location diagram (scale bar = 1 mm). (A) Representative image of microglial heterogenization at 3 and 7 days after ICH, CD16 (Red), CD206 (Green), Iba1 (Gray) (scale bar = 100 μm). (B) Representative images of Mac2‐positive microglia at 3, 7 days after ICH, Mac2 (Red), Iba1 (Gray) (scale bar = 100 μm). (C, D) Quantification of CD16‐positive and CD206‐positive microglia at 3 and 7 days after ICH. *n* = 3 for Sham; *n* = 5 for ICH and Vehicle, *n* = 5 for ICH and FK228. (E) The mRNA expression levels of INOS, Arg1, CD16, CD206 of microglia were detected by RT‐qPCR at 3 and 7 days after ICH. 3d, *n* = 4 for per group; 7d, *n* = 3 for Sham; *n* = 3 for ICH and Vehicle, *n* = 4 for ICH and FK228. (F) Quantification of Mac2‐positive microglia among three groups at 3 and 7 days after ICH. *n* = 3 for Sham; *n* = 5 for ICH and Vehicle, *n* = 5 for ICH and FK228. All the data are presented as the mean ± SD. One‐way ANOVA test and Bonferroni post hoc (C–F). *p < 0.05, **p < 0.01, ***p < 0.001, ns: no significance.

To substantiate the findings from the in vitro ICH model, we assessed the impact of FK228 on microglial heterogeneity in mice with ICH. In our investigation of ICH, we procured brain tissue at various intervals (3, 7 days post‐ICH) and subjected these samples to immunostaining, with the aim of validating the influence of FK228 on microglial heterogenization. Our analysis revealed a distinct shift in microglial expression patterns, transitioning from a high expression of CD16 to an elevated expression of CD206. Notably, the group administered FK228 demonstrated a significantly higher expression of CD206 compared to the vehicle‐treated group, suggesting that FK228 actively facilitates this phenotypic shift (Figure 4A,C,D). Additionally, the mRNA expression levels of Arg1 and CD206 were elevated in the group treated with FK228 after ICH (Figure 4E).

It's widely recognized that swiftly removing a hematoma is essential to reduce damage and improve clinical outcomes.^14^, ^53^ Therefore, to investigate the phagocytic phenotype of microglia and the FK228's effect on it post‐ICH, we employed co‐immunostaining for Mac‐2, a phagocytic biomarker at 1, 3, and 7 days following ICH (Figure S2B). Post‐ICH, a pronounced co‐localization of Mac‐2 with Iba‐1 was observed, indicating heightened phagocytic activity in microglia. Subsequently, our focus shifted to exploring the influence of FK228 on the phagocytic characteristics of microglia. The immunostaining results revealed that administration of FK228upregulated the expression of Mac‐2 (Figure 4B,F). Indeed, the heterogenization and phagocytic phenotype seems to be influenced after the administration of FK228.

**FIGURE 5:**
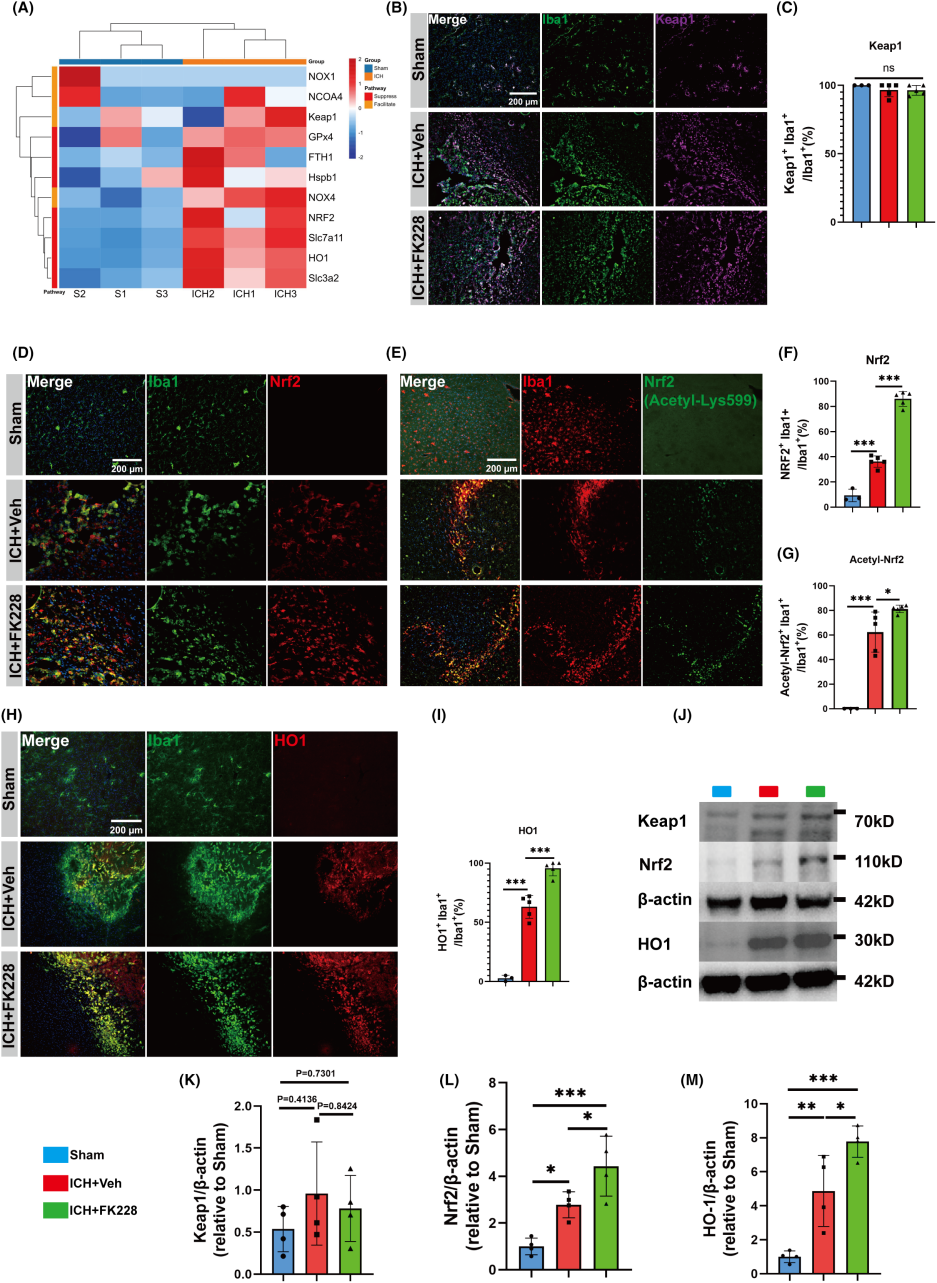
HDAC1/2 inhibition activates the Nrf2/HO1 signaling pathway in vivo ICH model. (A) Bioinformatics analysis of DEGs between ICH and control group. (B, C) Representative immunostaining image and quantification of Keap1 (purple) co‐localized with Iba1 (green) at 7 days after ICH in vivo. *n* = 3 for Sham; *n* = 5 for ICH and vehicle, *n* = 5 for ICH and FK228, (scale bar = 200 μm). (D, F) Representative immunostaining image and quantification of Nrf2 (red) co‐localized with Iba1 (green) at 7 days after ICH in vivo. *n* = 3 for Sham; *n* = 5 for ICH and vehicle, *n* = 5 for ICH and FK228, (scale bar = 200 μm). (E, G) Representative immunostaining image of Acetyl‐Nrf2 (green) co‐localized with Iba1 (red) at 7 days after ICH in vivo. *n* = 3 for Sham; *n* = 5 for ICH and vehicle, *n* = 5 for ICH and FK228, (scale bar = 200 μm). (H, I) Representative immunostaining image of HO1 (red) co‐localized with Iba1 (green) at 7 days after ICH in vivo. *n* = 3 for Sham; *n* = 5 for ICH and vehicle, *n* = 5 for ICH and FK228, (scale bar = 200 μm). (J) Western blotting analysis of Keap1/Nrf2/HO1 at 7 days after ICH *n* = 4 for Sham; *n* = 4 for ICH and vehicle, *n* = 4 for ICH and FK228. All the data are presented as the mean ± SD. One‐way ANOVA test and Bonferroni post hoc (A–M). *p < 0.05, **p < 0.01, ***p < 0.001, ns: no significance.

Investigations into the Keap1/Nrf2/HO1 signaling axis, a canonical antioxidant pathway, revealed its involvement in ferroptosis, particularly in lipid peroxide formation.^30^ In our exploration of the Keap1/Nrf2/HO1 signaling pathway's role in modulating oxidative stress within the ferroptosis and inflammation framework, we leveraged data from an ICH mouse model (GSE206971) comprising three ICH‐affected and three control mice.^54^ This analysis identified 11 differentially expressed genes (DEGs), including 9 upregulated and 2 downregulated genes. Notably, key components of this pathway Nrf2, and HO1were all upregulated post‐ICH (Figure 5A). These findings corroborate the activation of the Keap1/Nrf2/HO1 signaling pathway in response to ICH.

Normally, Nrf2 is sequestered in the cytoplasm by Keap1 protein, maintaining its inactive state. Keap1 promotes its degradation by directing Nrf2 into protein degradation pathways. Following ROS stimulation, the conformation of Keap1 undergoes changes, weakening its binding capacity with Nrf2. This allows Nrf2 to translocate into the nucleus, where it exerts its antioxidative stress function.^36^ Therefore, immunofluorescence staining to co‐localize Iba1 with Keap1 (Figure 5B), Nrf2 (Figure 5D), HO1 (Figure 5H) and the western blotting (Figure 5J) to examine the expression of the three molecular were conducted. The results revealed that the Keap1/Nrf2/HO1 signaling pathway was activated following ICH, indeed. Especially, the co‐expression levels of Nrf2 and HO1 with Iba1 were significantly upregulated when treated with FK228 (Figure 5F,I), and the protein expression levels of Nrf2 and HO1 were both upregulated (Figure 5L,M). However, the expression pattern of Keap1 seemed no difference among the three groups (Figure 5C,K).

Previous research has indicated that the acetylation of Nrf2 not only stabilizes it but also improves its capacity to penetrate the nucleus.^55^ Additionally, VPA upregulated the expression, activation and nuclear translocation of Nrf2 in mice heart tissue.^56^ Trichostatin A (TSA) lowered Keap1 expression, promoted Keap1/Nrf2 separation, Nrf2 nuclear translocation, and Nrf2's binding in HO1, potentially leading to Nrf2 acetylation.^37^ Therefore, we proposed that FK228 may regulate Nrf2 by increasing its acetylation levels. Immunofluorescence staining to colocalize Iba1 with acetyl‐Nrf2 (Acetyl‐Lys599) was conducted. The immunostaining results indicated that Nrf2 underwent acetylation in the microglia surrounding the hematoma following ICH (Figure 5E). Furthermore, the acetylation of Nrf2 seemed to be enhanced by FK228 (Figure 5G).

### 3.5 Inhibition of HDAC1/2 accelerates the clearance of hematoma and mitigates the neurological deficits in the in vivo ICH model

**FIGURE 6:**
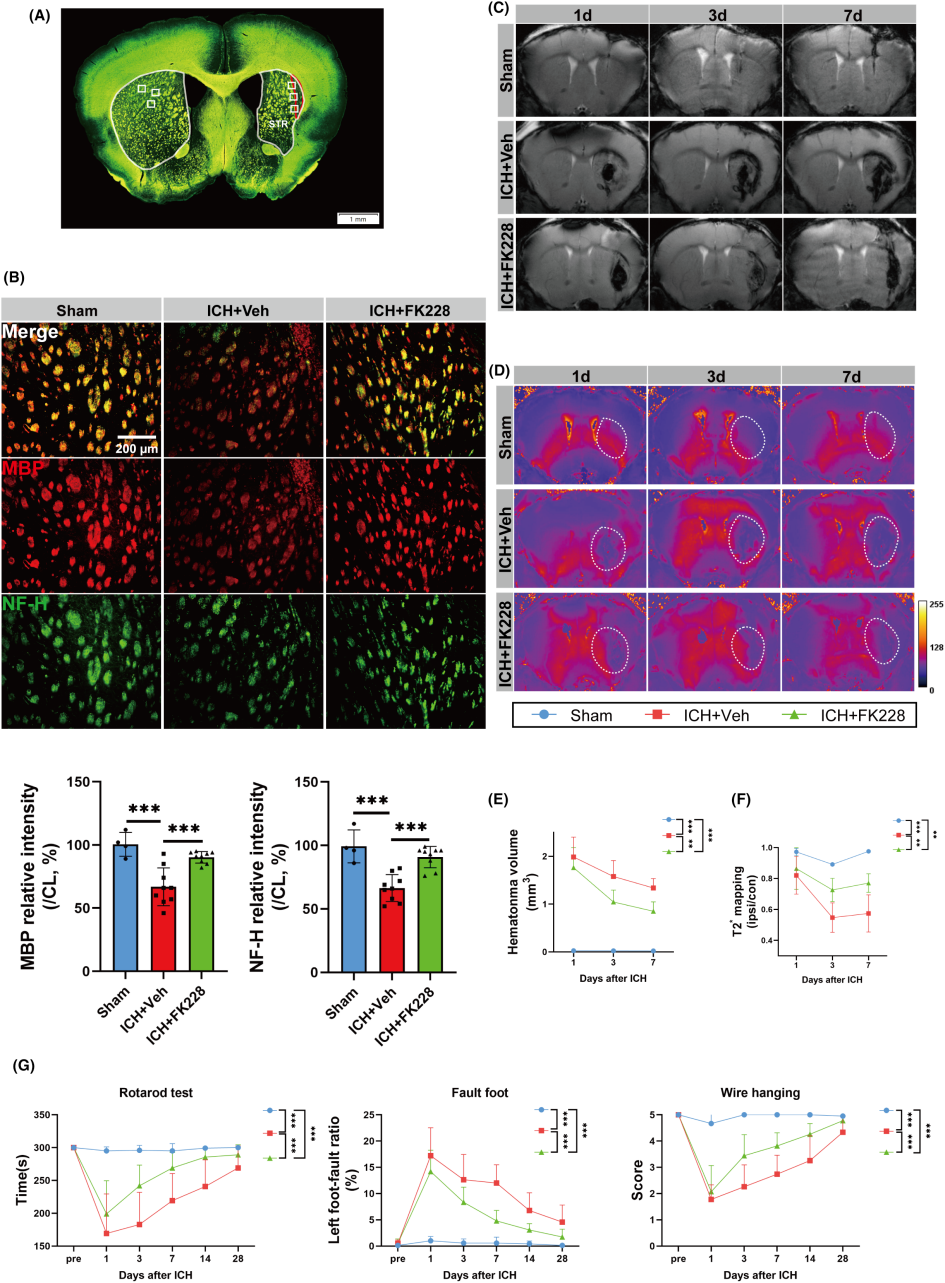
HDAC1/2 inhibition accelerates the clearance of hematoma and mitigates the neurological deficits in vivo ICH model. (A) Sampling location diagram (scale bar = 1 mm). (B) Representative immunostaining image and quantification of MBP (red) and NF‐H (green) at 35 days after ICH in vivo. *n* = 4 for Sham; *n* = 9 for ICH and Vehicle, *n* = 9 for ICH and FK228, (scale bar = 200 μm). (C, E) Coronal SWI images of radiological hematoma changes in mice after ICH, assessed by 11.7T Ultrahigh field magnetic resonance on the 1, 3 and 7 days after ICH. *n* = 3 for Sham; *n* = 4 for ICH and Vehicle, *n* = 4 for ICH and FK228. (D, F) Coronal T2* mapping images of radiological content of Iron in mice after ICH, assessed by 11.7T Ultrahigh field magnetic resonance on the 1, 3 and 7 days post‐ICH. *n* = 3 for Sham; *n* = 4 for ICH and Vehicle, *n* = 4 for ICH and FK228. (G) Fault foot, Rotarod and Wire hanging test for mice for up to 28 days after ICH in vivo, *n* = 7 for Sham; *n* = 14 for ICH and Vehicle, *n* = 15 for ICH and FK228 for Rotarod and Fault foot test; *n* = 7 for Sham; *n* = 9 for ICH and Vehicle, *n* = 9 for ICH and FK228 for Wire hanging test. All the data are presented as the mean ± SD. One‐way ANOVA test and Bonferroni post hoc (B), Two‐way ANOVA repeated measurement (E–G). **p < 0.01, ***p < 0.001, ns: no significance.

To determine the neuroprotective potential of FK228 in the ICH model, we performed a comprehensive analysis that included histological, radiological, and behavioral assessments Myelin Basic Protein (MBP) is essential in the myelination process and stabilizes the myelin sheath of neural fibers, while Neurofilament heavy protein (NF‐H), an intermediate filament in neurons, maintains axonal structure and function, supporting axonal shape and transport.^57^ Therefore, we analyzed MBP and NF‐H by immunofluorescence (Figure 6A). Our data indicated that ICH led to a decrease in both MBP and NF‐H fluorescence intensity in the striatum. This suggests a notable loss of myelin and severe axonal damage following ICH, treatment of FK228 alleviated theis damage (Figure 6B). Radiologically, we focused on the impact of FK228 on hematoma resolution post‐ICH. Employing SWI and T2* sequences, we captured temporal imaging data at 1, 3, and 7 days after ICH. The T2* sequence, sensitive to the magnetic relaxation properties of tissue, facilitated the assessment of iron accumulation around the hematoma. Intriguingly, SWI images revealed a consistent reduction in hematoma volume in FK228‐treated mice (Figure 6C,E). However, T2* mapping values exhibited a unique pattern, initially decreasing before increasing post‐ICH (Figure 6D,F), indicative of a dynamic iron mobilization process. Importantly, FK228 treatment was associated with lower T2* mapping values, suggesting its role in enhancing hematoma clearance and reducing iron deposition.

To assess the effect of FK228 on sensorimotor impairment post‐ICH, we conducted various behavioral evaluations, such as the rotarod, foot fault, and wire hanging tests following ICH.. Behaviorally, FK228 treatment correlated with notable improvements in various neurological tests. In the wire hanging test, FK228‐treated mice exhibited enhanced muscular strength with higher score. Similarly, Rotarod and fault‐foot tests demonstrated significant motor coordination enhancements in the treated group (Figure 6G).

## 4 DISCUSSION

Various Animal models of cerebrovascular diseases like ICH, TBI, and MCAO show ferroptosis.^8^, ^9^, ^10^ Inflammation is associated with the ferroptosis, and alleviating inflammation can further inhibit ferroptosis. Class I HDACs molecules regulate microglia, with simultaneous HDAC1/2 inhibition showing greater effects than targeting a single subtype.^11^, ^27^, ^58^ We demonstrated ferroptosis following ICH, highlighting its impact on lipid metabolism and neuronal death. Additionally, this research investigated the impact of the selective HDAC1/2 inhibitor, FK228, on microglial diversity and its capability to mitigate ferroptosis post‐ICH. The findings suggest FK228 as a potentially effective treatment for reducing hemorrhagic brain injury by alleviating both inflammation and ferroptosis.

Ferroptosis is defined by an increased build‐up of lipid peroxidation and reactive oxygen species originating from iron metabolism. Morphologically, it is characterized by unusually small mitochondria with denser mitochondrial membranes, and a reduction or disappearance of mitochondrial cristae.^7^, ^11^, ^13^, ^59^ Post ICH, brain injury is significantly caused by iron release from hemoglobin breakdown in hematomas, leading to ROS production. The presence of blood components from the hematoma activates immune cells, such as microglia, leading to an inflammatory response.^60^ HDAC1/2 have been found to enhance anti‐inflammatory microglia, aiding in inflammation inhibition. At present, we confirmed that inhibiting HDAC1/2 resulted in a significant upregulation of CD206 expression. This induction of microglia anti‐inflammatory heterogenization is considered beneficial for the injured brain. The reduction of ICH hematoma has been confirmed to correlate with improved long‐term outcomes.^61^, ^62^ At this context, we utilized the transwell system to search the link between the neuroinflammation and the ferroptosis‐neurons. The ROS, lipid droplet and the GPx4 was chosen to be the observation marks. It was discovered that employing FK228 in the study alleviated the ICH‐induced ferroptosis, marked by aberrant lipid metabolism and modulating microglial diversity.

Our study examined iron buildup, ROS generation, and lipid changes in vivo, crucial in ferroptosis and cell death. The Perl's stain findings revealed iron deposition around the peri‐hematoma tissue. Moreover, T2* mapping revealed that iron accumulation mainly occurs around the hematoma, not in its core, suggesting iron deposition may not solely stem from hemoglobin degradation. Beyond that, we inspected the situation of lipid metabolism continuously at 1, 3, and 7 days after ICH, the significant lipid droplet only can be seen at 7 days near hematoma following ICH. Moreover, we meticulously assessed the function and morphology of mitochondria. The most prominent and variable morphological alterations observed in ferroptosis include the diminution of mitochondrial size and the loss of cristae. Following ICH, a plethora of shrunken mitochondria have been detected within axonal structures.

The Keap1/Nrf2/HO1 signaling axis, recognized for its antioxidative and anti‐inflammatory properties, is well established.^63^, ^64^ TSA, an agent that inhibits histone deacetylases, downregulates the Nrf2 inhibitor Keap1, facilitating Nrf2's nuclear translocation and subsequent transcriptional activation of the antioxidative gene HO1.^37^ Furthermore, the RNA‐sequence data indicated that the expression of Nrf2 and HO1 was significantly increased after ICH. In the present study, the expression of both also elevated highly. Besides, we verified that Keap1, Nrf2, and HO1 are co‐localized with microglia, suggesting their role in antioxidative activities within microglia. The expression level of Keap1 had not change. Additionally, Nrf2 undergoes various post‐translational modifications, including phosphorylation, ubiquitination, and acetylation. Currently, the phosphorylation modification of NRF2 is a widely studied area. Protein kinase C, casein kinase 2, AMP‐activated kinase, and GSK‐3 regulate the activity of NRF2 through phosphorylation modifications, playing a role in its physiological process against oxidative stress.^65^, ^66^ A small number of studies have reported that Nrf2 acetylation hinders its recognition by Keap1 and subsequent ubiquitination, reducing proteasomal degradation and enhancing Nrf2 stability.^38^, ^55^ However, research on the acetylation modification of Nrf2 is still relatively scarce, involving a limited range of diseases and cell types. In our study, we found that the acetylation of Nrf2 occurred in microglia and observed increased acetylation of Nrf2 in mice treated with FK228. This suggests that FK228 might activate the Nrf2‐HO1 signaling pathway by altering Nrf2's acetylation levels. Certainly, Nrf2 may also exert its anti‐ferroptosis effects by activating other pathways, such as upregulating GPx4, which we did not explore in this study.^67^

## 5 CONCLUSION

Our study shows that HDAC1/2 inhibition reduces neuro‐ferroptosis by affecting microglial heterogenization. A key finding is the crucial role of the Nrf2/HO1 pathway, particularly acetyl‐Nrf2, activated by HDAC1/2 inhibition, leading to improved neurological recovery post‐ICH.

## AUTHOR CONTRIBUTIONS

Yuxiang Gu and Yanqin Gao designed this study. Zhiwen Jiang, Heng Yang, Xu Pei, Jiabin Su, Hanqiang Jiang, Ruiyuan Weng, Yuchao Fei, and Xinjie Gao performed experiments. Zhiwen Jiang and Xinjie Gao analyzed the data. Zhiwen Jiang, Heng Yang, and Wei Ni wrote the manuscript. Yuxiang Gu and Yanqin Gao critically edited the manuscript. The authors read and approved the final manuscript.

## FUNDING INFORMATION

This work was supported by STI 2030‐Major Projects (2022ZD0204704, 2021ZD0201704), and National Natural Science Foundation of China (82,271,338 and 82,101,397), Shanghai Shenkang Hospital Development Center (SHDC2020CR1018B), Shanghai Municipal Science and Technology Major Project (2018SHZDZX03, 2018SHZDZX01), National Key R&D Program of China (2016YFC1300800), ZJLab, and Shanghai Center for Brain Science and Brain‐Inspired Technology.

## CONFLICT OF INTEREST STATEMENT

All authors declare that they have no competing interests.

## Supporting information


Data S1.

## Data Availability

The data used to support the findings of this study are available from the corresponding author upon request.

## References

[1] GBD 2019 Stroke Collaborators . Global, regional, and national burden of stroke and its risk factors, 1990–2019: a systematic analysis for the Global Burden of Disease Study 2019. Lancet Neurol. 2021;20(10):795‐820. doi:10.1016/S1474-4422(21)00252-0 34487721 PMC8443449

[2] Xiong XY , Wang J , Qian ZM , Yang QW . Iron and intracerebral hemorrhage: from mechanism to translation. Transl Stroke Res. 2014;5(4):429‐441. 24362931 10.1007/s12975-013-0317-7

[3] Xi G , Strahle J , Hua Y , Keep RF . Progress in translational research on intracerebral hemorrhage: is there an end in sight? Prog Neurobiol. 2014;115:45‐63. 24139872 10.1016/j.pneurobio.2013.09.007 PMC3961535

[4] Wan Y , Holste KG , Hua Y , Keep RF , Xi G . Brain edema formation and therapy after intracerebral hemorrhage. Neurobiol Dis. 2023;176:105948. 36481437 10.1016/j.nbd.2022.105948 PMC10013956

[5] Shah R , Shchepinov MS , Pratt DA . Resolving the role of lipoxygenases in the initiation and execution of ferroptosis. ACS Cent Sci. 2018;4(3):387‐396. 29632885 10.1021/acscentsci.7b00589 PMC5879472

[6] Wenzel SE , Tyurina YY , Zhao J , et al. PEBP1 wardens ferroptosis by enabling lipoxygenase generation of lipid death signals. Cell. 2017;171(3):628‐641 e626. 29053969 10.1016/j.cell.2017.09.044 PMC5683852

[7] Dixon SJ , Lemberg KM , Lamprecht MR , et al. Ferroptosis: an iron‐dependent form of nonapoptotic cell death. Cell. 2012;149(5):1060‐1072. 22632970 10.1016/j.cell.2012.03.042 PMC3367386

[8] Li Q , Han X , Lan X , et al. Inhibition of neuronal ferroptosis protects hemorrhagic brain. JCI Insight. 2017;2(7):e90777. 28405617 10.1172/jci.insight.90777 PMC5374066

[9] Xie BS , Wang YQ , Lin Y , et al. Inhibition of ferroptosis attenuates tissue damage and improves long‐term outcomes after traumatic brain injury in mice. CNS Neurosci Ther. 2019;25(4):465‐475. 30264934 10.1111/cns.13069 PMC6488926

[10] Tuo QZ , Lei P , Jackman KA , et al. Tau‐mediated iron export prevents ferroptotic damage after ischemic stroke. Mol Psychiatry. 2017;22(11):1520‐1530. 28886009 10.1038/mp.2017.171

[11] Rroji O , Kumar A , Karuppagounder SS , Ratan RR . Epigenetic regulators of neuronal ferroptosis identify novel therapeutics for neurological diseases: HDACs, transglutaminases, and HIF prolyl hydroxylases. Neurobiol Dis. 2021;147:105145. 33127469 10.1016/j.nbd.2020.105145 PMC7797188

[12] Jiang X , Stockwell BR , Conrad M . Ferroptosis: mechanisms, biology and role in disease. Nat Rev Mol Cell Biol. 2021;22(4):266‐282. 33495651 10.1038/s41580-020-00324-8 PMC8142022

[13] Xie Y , Hou W , Song X , et al. Ferroptosis: process and function. Cell Death Differ. 2016;23(3):369‐379. 26794443 10.1038/cdd.2015.158 PMC5072448

[14] Chang CF , Wan J , Li Q , Renfroe SC , Heller NM , Wang J . Alternative activation‐skewed microglia/macrophages promote hematoma resolution in experimental intracerebral hemorrhage. Neurobiol Dis. 2017;103:54‐69. 28365213 10.1016/j.nbd.2017.03.016 PMC5540140

[15] Chen B , Xie C , Shi T , et al. Activation of Swell1 in microglia suppresses neuroinflammation and reduces brain damage in ischemic stroke. Neurobiol Dis. 2023;176:105936. 36511337 10.1016/j.nbd.2022.105936

[16] Cao C , Ding J , Cao D , et al. TREM2 modulates neuroinflammation with elevated IRAK3 expression and plays a neuroprotective role after experimental SAH in rats. Neurobiol Dis. 2022;171:105809. 35781003 10.1016/j.nbd.2022.105809

[17] Wang J . Preclinical and clinical research on inflammation after intracerebral hemorrhage. Prog Neurobiol. 2010;92(4):463‐477. 20713126 10.1016/j.pneurobio.2010.08.001 PMC2991407

[18] Zhang Z , Zhang Z , Lu H , Yang Q , Wu H , Wang J . Microglial polarization and inflammatory mediators after intracerebral hemorrhage. Mol Neurobiol. 2017;54(3):1874‐1886. 26894396 10.1007/s12035-016-9785-6 PMC4991954

[19] Lan X , Han X , Li Q , Yang QW , Wang J . Modulators of microglial activation and polarization after intracerebral haemorrhage. Nat Rev Neurol. 2017;13(7):420‐433. 28524175 10.1038/nrneurol.2017.69 PMC5575938

[20] Marks PA , Miller T , Richon VM . Histone deacetylases. Curr Opin Pharmacol. 2003;3(4):344‐351. 12901942 10.1016/s1471-4892(03)00084-5

[21] Yang H , Ni W , Wei P , et al. HDAC inhibition reduces white matter injury after intracerebral hemorrhage. J Cereb Blood Flow Metab. 2021;41(5):958‐974. 32703113 10.1177/0271678X20942613 PMC8054714

[22] Zhao Y , Mu H , Huang Y , et al. Microglia‐specific deletion of histone deacetylase 3 promotes inflammation resolution, white matter integrity, and functional recovery in a mouse model of traumatic brain injury. J Neuroinflammation. 2022;19(1):201. 35933343 10.1186/s12974-022-02563-2 PMC9357327

[23] Patnala R , Arumugam TV , Gupta N , Dheen ST . HDAC inhibitor sodium butyrate‐mediated epigenetic regulation enhances neuroprotective function of microglia during ischemic stroke. Mol Neurobiol. 2017;54(8):6391‐6411. 27722928 10.1007/s12035-016-0149-z

[24] Park MJ , Sohrabji F . The histone deacetylase inhibitor, sodium butyrate, exhibits neuroprotective effects for ischemic stroke in middle‐aged female rats. J Neuroinflammation. 2016;13(1):300. 27905989 10.1186/s12974-016-0765-6 PMC5131416

[25] Wang G , Shi Y , Jiang X , et al. HDAC inhibition prevents white matter injury by modulating microglia/macrophage polarization through the GSK3beta/PTEN/Akt axis. Proc Natl Acad Sci USA. 2015;112(9):2853‐2858. 25691750 10.1073/pnas.1501441112 PMC4352818

[26] Kannan V , Brouwer N , Hanisch UK , Regen T , Eggen BJ , Boddeke HW . Histone deacetylase inhibitors suppress immune activation in primary mouse microglia. J Neurosci Res. 2013;91(9):1133‐1142. 23686642 10.1002/jnr.23221

[27] Jeong Y , Du R , Zhu X , et al. Histone deacetylase isoforms regulate innate immune responses by deacetylating mitogen‐activated protein kinase phosphatase‐1. J Leukoc Biol. 2014;95(4):651‐659. 24374966 10.1189/jlb.1013565

[28] Datta M , Staszewski O , Raschi E , et al. Histone deacetylases 1 and 2 regulate microglia function during development, homeostasis, and neurodegeneration in a context‐dependent manner. Immunity. 2018;48(3):514‐529 e516. 29548672 10.1016/j.immuni.2018.02.016

[29] Kong Q , Li F , Sun K , Sun X , Ma J . Valproic acid ameliorates cauda equina injury by suppressing HDAC2‐mediated ferroptosis. CNS Neurosci Ther. 2023. doi:10.1111/cns.14524. Epub ahead of print. PMC11017456 38105511

[30] Dodson M , Castro‐Portuguez R , Zhang DD . NRF2 plays a critical role in mitigating lipid peroxidation and ferroptosis. Redox Biol. 2019;23:101107. 30692038 10.1016/j.redox.2019.101107 PMC6859567

[31] Li C , Wu Z , Xue H , et al. Ferroptosis contributes to hypoxic‐ischemic brain injury in neonatal rats: role of the SIRT1/Nrf2/GPx4 signaling pathway. CNS Neurosci Ther. 2022;28(12):2268‐2280. 36184790 10.1111/cns.13973 PMC9627393

[32] Chen Y , He W , Wei H , et al. Srs11‐92, a ferrostatin‐1 analog, improves oxidative stress and neuroinflammation via Nrf2 signal following cerebral ischemia/reperfusion injury. CNS Neurosci Ther. 2023;29(6):1667‐1677. 36852441 10.1111/cns.14130 PMC10173707

[33] Ding P , Chen W , Yan X , et al. BMPER alleviates ischemic brain injury by protecting neurons and inhibiting neuroinflammation via Smad3‐Akt‐Nrf2 pathway. CNS Neurosci Ther. 2022;28(4):593‐607. 34904361 10.1111/cns.13782 PMC8928915

[34] Che J , Wang H , Dong J , et al. Human umbilical cord mesenchymal stem cell‐derived exosomes attenuate neuroinflammation and oxidative stress through the NRF2/NF‐kappaB/NLRP3 pathway. CNS Neurosci Ther. 2023. doi:10.1111/cns.14454. Epub ahead of print. PMC10916441 37697971

[35] Zhang Y , Ye P , Zhu H , et al. Neutral polysaccharide from Gastrodia elata alleviates cerebral ischemia‐reperfusion injury by inhibiting ferroptosis‐mediated neuroinflammation via the NRF2/HO‐1 signaling pathway. CNS Neurosci Ther. 2023. doi:10/1111/cns.14456. Epub ahead of print. 10.1111/cns.14456 PMC10916450 37752806

[36] Kansanen E , Kuosmanen SM , Leinonen H , Levonen AL . The Keap1‐Nrf2 pathway: mechanisms of activation and dysregulation in cancer. Redox Biol. 2013;1(1):45‐49. 24024136 10.1016/j.redox.2012.10.001 PMC3757665

[37] Wang B , Zhu X , Kim Y , et al. Histone deacetylase inhibition activates transcription factor Nrf2 and protects against cerebral ischemic damage. Free Radic Biol Med. 2012;52(5):928‐936. 22226832 10.1016/j.freeradbiomed.2011.12.006 PMC6010182

[38] Ganner A , Pfeiffer ZC , Wingendorf L , et al. The acetyltransferase p300 regulates NRF2 stability and localization. Biochem Biophys Res Commun. 2020;524(4):895‐902. 32057361 10.1016/j.bbrc.2020.02.006

[39] West AC , Johnstone RW . New and emerging HDAC inhibitors for cancer treatment. J Clin Invest. 2014;124(1):30‐39. 24382387 10.1172/JCI69738 PMC3871231

[40] Han Z , Zhao H , Tao Z , et al. TOPK promotes microglia/macrophage polarization towards M2 phenotype via inhibition of HDAC1 and HDAC2 activity after transient cerebral ischemia. Aging Dis. 2018;9(2):235‐248. 29896413 10.14336/AD.2017.0328 PMC5963345

[41] Zille M , Kumar A , Kundu N , et al. Ferroptosis in neurons and cancer cells is similar but differentially regulated by histone deacetylase inhibitors. eNeuro. 2019;6(1):ENEURO.0263‐18.2019. 10.1523/ENEURO.0263-18.2019 PMC6378329 30783618

[42] Gao X , Yang H , Xiao W , et al. Modified exosomal SIRPalpha variants alleviate white matter injury after intracerebral hemorrhage via microglia/macrophages. Biomater Res. 2022;26(1):67. 36435797 10.1186/s40824-022-00311-4 PMC9701394

[43] Dharmalingam P , Talakatta G , Mitra J , et al. Pervasive genomic damage in experimental intracerebral hemorrhage: therapeutic potential of a mechanistic‐based carbon nanoparticle. ACS Nano. 2020;14(3):2827‐2846. 32049495 10.1021/acsnano.9b05821 PMC7850811

[44] Okabe S , Tauchi T , Nakajima A , et al. Depsipeptide (FK228) preferentially induces apoptosis in BCR/ABL‐expressing cell lines and cells from patients with chronic myelogenous leukemia in blast crisis. Stem Cells Dev. 2007;16(3):503‐514. 17610380 10.1089/scd.2007.9994

[45] Ge MH , Tian H , Mao L , et al. Zinc attenuates ferroptosis and promotes functional recovery in contusion spinal cord injury by activating Nrf2/GPX4 defense pathway. CNS Neurosci Ther. 2021;27(9):1023‐1040. 33951302 10.1111/cns.13657 PMC8339532

[46] Wu H , Wu T , Xu X , Wang J , Wang J . Iron toxicity in mice with collagenase‐induced intracerebral hemorrhage. J Cereb Blood Flow Metab. 2011;31(5):1243‐1250. 21102602 10.1038/jcbfm.2010.209 PMC3099628

[47] Klasson TD , LaGory EL , Zhao H , et al. ACSL3 regulates lipid droplet biogenesis and ferroptosis sensitivity in clear cell renal cell carcinoma. Cancer Metab. 2022;10(1):14. 36192773 10.1186/s40170-022-00290-z PMC9528056

[48] Li LH , Zhang PR , Cai PY , Li ZC . Histone deacetylase inhibitor, Romidepsin (FK228) inhibits endometrial cancer cell growth through augmentation of p53‐p21 pathway. Biomed Pharmacother. 2016;82:161‐166. 27470351 10.1016/j.biopha.2016.04.053

[49] Ni M , Esposito E , Raj VP , et al. New macrocyclic analogs of the natural histone deacetylase inhibitor FK228; design, synthesis and preliminary biological evaluation. Bioorg Med Chem. 2015;23(21):6785‐6793. 26481659 10.1016/j.bmc.2015.10.004

[50] Hoshino I , Matsubara H , Hanari N , et al. Histone deacetylase inhibitor FK228 activates tumor suppressor Prdx1 with apoptosis induction in esophageal cancer cells. Clin Cancer Res. 2005;11(21):7945‐7952. 16278420 10.1158/1078-0432.CCR-05-0840

[51] Yan N , Xu Z , Qu C , Zhang J . Dimethyl fumarate improves cognitive deficits in chronic cerebral hypoperfusion rats by alleviating inflammation, oxidative stress, and ferroptosis via NRF2/ARE/NF‐kappaB signal pathway. Int Immunopharmacol. 2021;98:107844. 34153667 10.1016/j.intimp.2021.107844

[52] Gao M , Yi J , Zhu J , et al. Role of mitochondria in ferroptosis. Mol Cell. 2019;73(2):354‐363 e353. 30581146 10.1016/j.molcel.2018.10.042 PMC6338496

[53] Wilkinson DA , Keep RF , Hua Y , Xi G . Hematoma clearance as a therapeutic target in intracerebral hemorrhage: from macro to micro. J Cereb Blood Flow Metab. 2018;38(4):741‐745. 29350086 10.1177/0271678X17753590 PMC5888862

[54] Zheng Y , Fan L , Xia S , et al. Role of complement C1q/C3‐CR3 signaling in brain injury after experimental intracerebral hemorrhage and the effect of minocycline treatment. Front Immunol. 2022;13:919444. 36189326 10.3389/fimmu.2022.919444 PMC9520460

[55] Kawai Y , Garduno L , Theodore M , Yang J , Arinze IJ . Acetylation‐deacetylation of the transcription factor Nrf2 (nuclear factor erythroid 2‐related factor 2) regulates its transcriptional activity and nucleocytoplasmic localization. J Biol Chem. 2011;286(9):7629‐7640. 21196497 10.1074/jbc.M110.208173 PMC3045017

[56] Lei I , Huang W , Noly PE , et al. Metabolic reprogramming by immune‐responsive gene 1 up‐regulation improves donor heart preservation and function. Sci Transl Med. 2023;15(682):eade3782. 36753565 10.1126/scitranslmed.ade3782 PMC10068866

[57] Wang KK , Yang Z , Zhu T , et al. An update on diagnostic and prognostic biomarkers for traumatic brain injury. Expert Rev Mol Diagn. 2018;18(2):165‐180. 29338452 10.1080/14737159.2018.1428089 PMC6359936

[58] Oliveira T , Hermann E , Lin D , Chowanadisai W , Hull E , Montgomery M . HDAC inhibition induces EMT and alterations in cellular iron homeostasis to augment ferroptosis sensitivity in SW13 cells. Redox Biol. 2021;47:102149. 34600336 10.1016/j.redox.2021.102149 PMC8487084

[59] Oun A , Soliman A , Trombetta‐Lima M , et al. LRRK2 protects immune cells against erastin‐induced ferroptosis. Neurobiol Dis. 2022;175:105917. 36336242 10.1016/j.nbd.2022.105917

[60] Magid‐Bernstein J , Girard R , Polster S , et al. Cerebral hemorrhage: pathophysiology, treatment, and future directions. Circ Res. 2022;130(8):1204‐1229. 35420918 10.1161/CIRCRESAHA.121.319949 PMC10032582

[61] Hanley DF , Thompson RE , Rosenblum M , et al. Efficacy and safety of minimally invasive surgery with thrombolysis in intracerebral haemorrhage evacuation (MISTIE III): a randomised, controlled, open‐label, blinded endpoint phase 3 trial. Lancet. 2019;393(10175):1021‐1032. 30739747 10.1016/S0140-6736(19)30195-3 PMC6894906

[62] Lu J , Li Z , Zhao Q , Liu D , Mei YA . Neuritin improves the neurological functional recovery after experimental intracerebral hemorrhage in mice. Neurobiol Dis. 2021;156:105407. 34058347 10.1016/j.nbd.2021.105407

[63] Vomund S , Schafer A , Parnham MJ , Brune B , von Knethen A . Nrf2, the master regulator of anti‐oxidative responses. Int J Mol Sci. 2017;18(12):2772. 29261130 10.3390/ijms18122772 PMC5751370

[64] Loboda A , Damulewicz M , Pyza E , Jozkowicz A , Dulak J . Role of Nrf2/HO‐1 system in development, oxidative stress response and diseases: an evolutionarily conserved mechanism. Cell Mol Life Sci. 2016;73(17):3221‐3247. 27100828 10.1007/s00018-016-2223-0 PMC4967105

[65] Liu T , Lv YF , Zhao JL , You QD , Jiang ZY . Regulation of Nrf2 by phosphorylation: consequences for biological function and therapeutic implications. Free Radic Biol Med. 2021;168:129‐141. 33794311 10.1016/j.freeradbiomed.2021.03.034

[66] Fao L , Mota SI , Rego AC . Shaping the Nrf2‐ARE‐related pathways in Alzheimer's and Parkinson's diseases. Ageing Res Rev. 2019;54:100942. 31415806 10.1016/j.arr.2019.100942

[67] Yang B , Pan J , Zhang XN , et al. NRF2 activation suppresses motor neuron ferroptosis induced by the SOD1(G93A) mutation and exerts neuroprotection in amyotrophic lateral sclerosis. Neurobiol Dis. 2023;184:106210. 37352984 10.1016/j.nbd.2023.106210

