# Supplementary material for: Attenuation of neuronal ferroptosis in intracerebral hemorrhage by inhibiting HDAC1/2: Microglial heterogenization via the Nrf2/HO1 pathway
Source: CNS Neurosci Ther. 2024 Mar 24;30(3):e14646. doi: 10.1111/cns.14646 (PMC10961428; doi:10.1111/cns.14646)
Supplement: Supplementary file 1 — Data S1. [file CNS-30-e14646-s001.pdf]

Attenuation of Neuronal Ferroptosis in Intracerebral hemorrhage by inhibiting HDAC1/2: Microglial Heterogenization via the Nrf2/HO1 Pathway

Zhiwen Jiang*, Heng Yang*, Wei Ni, Xinjie Gao, Hanqiang Jiang, Jiabin Su, Ruiyuan Weng, Yuchao Fei, Yanqin Gao, Yuxiang Gu

Department of Neurosurgery of Huashan Hospital, State Key Laboratory of Medical Neurobiology and MOE Frontiers Center for Brain Science, Institutes of Brain Science, Fudan University, Shanghai 200032, China

*Zhiwen Jiang and Heng Yang contributed equally to this work

Address Correspondence to

Dr. Yuxiang Gu,

Department of Neurosurgery of Huashan Hospital, State Key Laboratory of Medical Neurobiology and MOE Frontiers Center for Brain Science, Institutes of Brain Science, Fudan University, Shanghai 200032, China

E-mail address: guyuxiang1972@163.com

Or

Dr. Yanqin Gao,

Department of Neurosurgery of Huashan Hospital, State Key Laboratory of Medical Neurobiology and MOE Frontiers Center for Brain Science, Institutes of Brain Science, Fudan University, Shanghai 200032, China

E-mail address: yqgao@shmu.edu.cn

Supplementary materials

Table S1 Primer sequences used in real-time PCR analysis

Primer		Sequence
CD16	F	5'-ACACATGTTCTCTGGGAAATC-3'
	R	5'-AGTGCATCATCGTTGTTCATA-3'
INOS	F	5'-CAAGCACCTTGGAAGAGGAG-3'
	R	5'-CCTTTCAGTCCTTTGCAAGC-3'
GAPDH	F	5'-CTGCCCAGAACATCATCCCT-3'
	R	5'-TGAAGTCGCAGGAGACAACC-3'
GPx4	F	5'-CCCGATATGCTGAGTGTGGTTTAC-3'
	R	5'-TTTCTTGATTACTTCCTGGCTCCTG-3'
FTH1	F	5'-CCATCAACCGCCAGATCAACC-3'
	R	5'-CAAAGTTCTTCAGAGCCACATCATC-3'
ACSL4	F	5'-CTATGGCTGTAGGATTGGATACTCTTC-3'
	R	5'-AATCACCCTTGCTTCCCTTCTTG-3'
IL10	F	5'-AGAGAAGCATGGCCCAGAAATCAAG-3'
	R	5'-CTTCACCTGCTCCACTGCCTTG-3'
IL6	F	5'-GAAACCGCTATGAAGTTCCTCTCTG-3'
	R	5'-GTATCCTCTGTGAAGTCTCCTCTCC-3'
TNF-α	F	5'-CGCTCTTCTGTCTACTGAACTTCGG-3'
	R	5'-GTGGTTTGTGAGTGTGAGGGTCTG-3'
TGF-β	F	5'-CTCCCGTGGCTTCTAGTGC-3'
	R	5'-GCCTTAGTTTGGACAGGATCTG-3'
Arg1	F	5'-CTCCAAGCCAAAGTCCTTAGAG-3'
	R	5'-AGGAGCTGTCATTAGGGACATC-3'
CD206	F	5'-GTCTGAGTGTACGCAGTGGTTGG-3'
	R	5'-TCTGATGATGGACTTCCTGGTAGCC-3'

Supplementary Figures

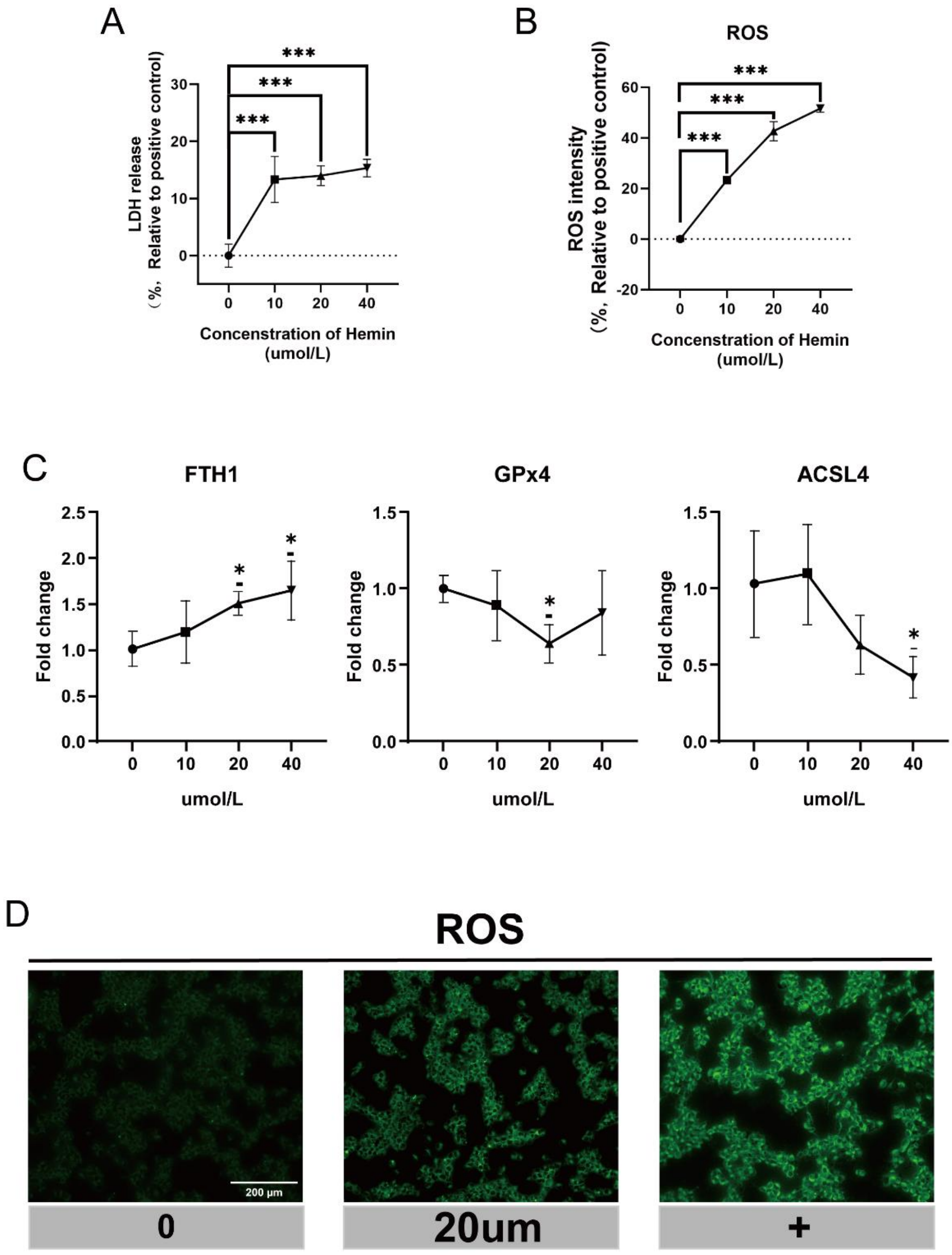

Figure S1 Ferroptosis phenotype in vitro ICH model. (A) LDH release assay across a Hemin concentration gradient (0 10, 20, 40umol) after 24 hours. (B) Reactive oxygen species (ROS) levels detection in HT22 cells after 24 hours of exposure to increasing Hemin concentrations, highlighting oxidative stress. (C) The mRNA expression levels of FTH1, GPx4, ACSL4 as quantified by RT-qPCR following Hemin treatment. (D)

Representative images of ROS levels detection in HT22 cells after 24 hours of exposure to Hemin(20umol) (scale bar = 200um). All the data are presented as the mean ± SD. One-way ANOVA test and Bonferroni post hoc (A-C). * p<0.05, *** p<0.001, ns: no significance.

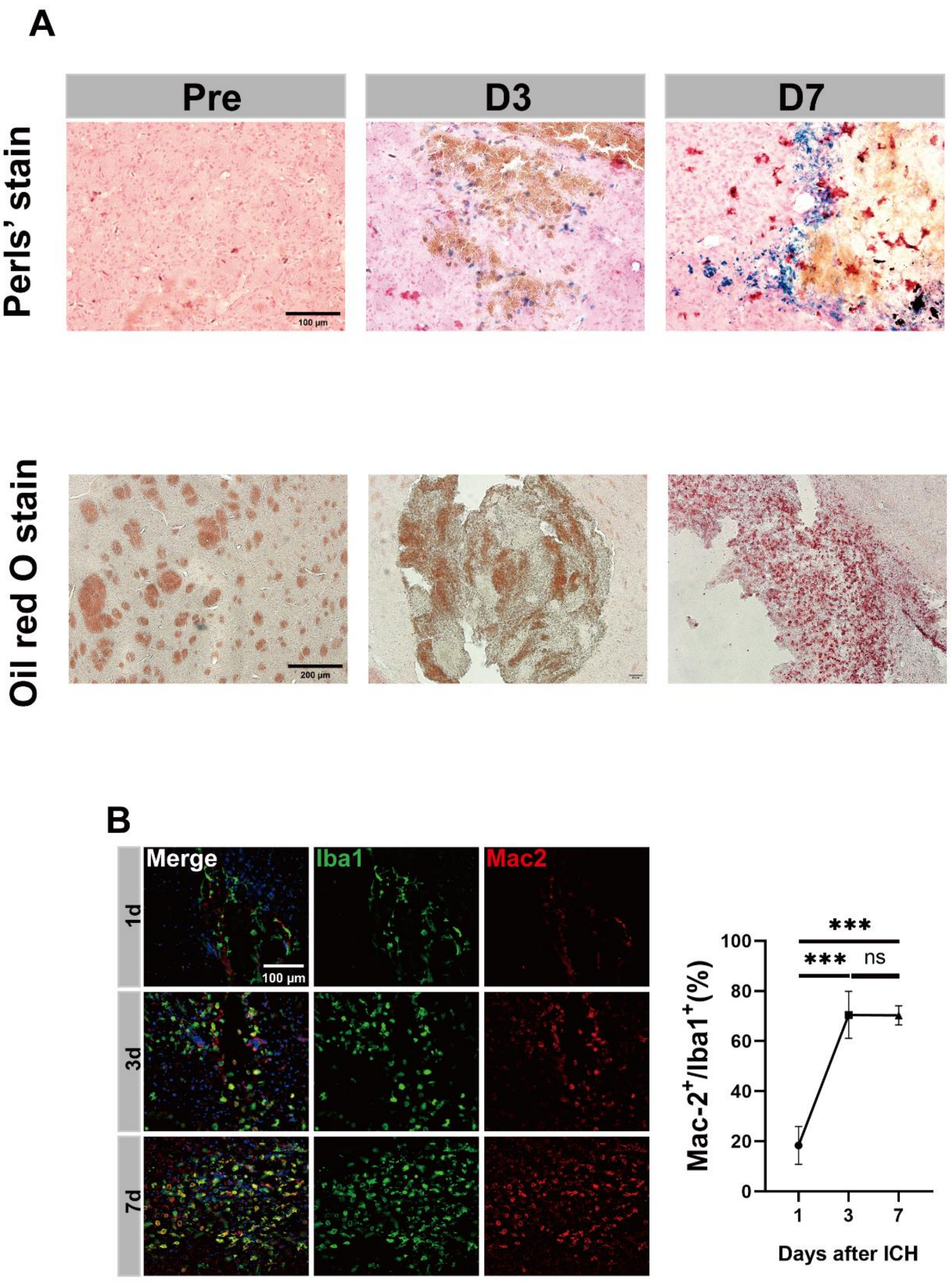

Figure S2 Ferroptosis phenotype and microglial phagocytic phenotype after ICH in vivo. (A) Perls' stain and Oil red O staining at 3 and 7days after ICH (scale bar1 = 100um, scale bar2 = 200um,). (B) Representative images of Mac2(red) and Iba1(green) immunostaining at 1, 3 and 7days after ICH (scale bar = 100um) All the data are presented as the mean ±

SD. One-way ANOVA test and Bonferroni post hoc (B). *** p<0.001, ns: no significance.

Supplementary Methods

Immunofluorescence

Anesthetized mice underwent transcardial perfusion with PBS and 4% paraformaldehyde for brain fixation. Brains were then immersed in 4% paraformaldehyde and 20% and 30% sucrose solutions for fixation and dehydration. 25-μm coronal sections were cut using a freezing microtome (HM525NX, TermoFisher, USA). Sections were washed and blocked with 10% goat or donkey serum for 1 hour. Subsequently, the brain sections were incubated overnight at 4°C with the following primary antibodies: anti-NeuN (1:1000, ab177487, abcam), anti-Iba1 (1:1000, ab5076, abcam), anti-CD16/32 (1:200, ab25235, abcam), anti-CD206 (1:200, 17–7321-82, R&D), anti-Mac2 (1:500, AF1197, R&D), anti-Keap1 (1:1,000, 10503-2-AP, Protein-tech), anti-Nrf2 (1:1,000, 16369-1-AP, Protein-tech), anti-Nrf2(Acetyl-Lys599) (1:500 HW147, Signalway antibody), anti-HO1 (1:1,000 16369-1-AP, Protein-tech), anti-MBP (1:500, ab40390, abcam), anti-NF-H (1:200, ab82259, abcam). Following the washing step, brain slices were incubated for one hour at room temperature with suitable Alexa-Fluor-labeled secondary antibodies (dilution 1:1000; sourced from Jackson ImmunoResearch, USA).

Perl's blue stain

3,3′-Diaminobenzidine (DAB; Vector Laboratories)-enhanced Perls' staining was used to detect iron accumulation as previously described.[37] Briefly, sections of brain tissue were washed with PBS and incubated in freshly prepared Perls' solution (5% potassium ferrocyanide [Sigma-Aldrich]/10% hydrochloric acid) for 1 hour, followed by 5 PBS washes. After DAB incubation for 3 minutes and hematoxylin (Sigma-Aldrich) counterstaining, iron deposition was digitized and analyzed with ImageJ software.

Oil red O assay

Oil Red O staining was performed to assess lipid deposition in the peri-hematoma area tissue using Modified Oil Red O Staining Kit (Beyotime, Shanghai, China). The

cryosections of brain tissues (25 μm) were stained with 0.5% Oil Red O for 15 min. Then, the sections were observed under an optical microscope.
